# Peripheral blood HIF-1α levels in type 2 diabetes mellitus and their association with diabetic complications: a systematic review and meta-analysis

**DOI:** 10.3389/fendo.2026.1879557

**Published:** 2026-07-29

**Authors:** Yi Su, Yunhui Chen, Heng Yang, Qingzhi Liang, Ting Luo, Yulin Leng, Jiahong Zhang, Ting Zheng, Chunguang Xie, Xiaoxu Fu

**Affiliations:** 1 Chengdu University of Traditional Chinese Medicine, Chengdu, Sichuan, China; 2 School of Clinical Medicine, Chengdu University of Traditional Chinese Medicine, Chengdu, Sichuan, China; 3 Department of Dermatology, Hospital of Chengdu University of Traditional Chinese Medicine, Chengdu, Sichuan, China; 4 Hospital of Chengdu University of Traditional Chinese Medicine, Chengdu, Sichuan, China; 5 Traditional Chinese Medicine (TCM) Prevention and Treatment of Metabolic and Chronic Diseases Key Laboratory of Sichuan Province, Hospital of Chengdu University of Traditional Chinese Medicine, Chengdu, Sichuan, China; 6 Department of Endocrinology, Hospital of Chengdu University of Traditional Chinese Medicine, Chengdu, Sichuan, China; 7 Zhongshan Hospital Affiliated to Dalian University, Dalian, Liaoning, China

**Keywords:** diabetic complications, hypoxia-inducible factor-1alpha, meta-analysis, type 2 diabetes mellitus, vascular endothelial growth factor

## Abstract

**Background:**

Hyperglycemia is known to exacerbate cellular hypoxia. Hypoxia-inducible factor-1α (HIF-1α) is the functional subunit of the HIF-1 transcription factor and plays a central role in regulating cellular adaptive responses to low oxygen tension. HIF-1α orchestrates these adaptive responses by regulating downstream effectors such as vascular endothelial growth factor (VEGF), which is essential for vascular integrity and metabolic homeostasis. However, while these molecular pathways are well-defined *in vitro*, there is a lack of a comprehensive synthesis of their circulating levels in type 2 diabetes mellitus (T2DM) patients. This gap in systemic evidence necessitates a rigorous meta-analysis to provide a definitive quantitative assessment.

**Methods:**

We conducted a systematic search of major English and Chinese databases (PubMed, Embase, Cochrane Library, Web of Science, Wanfang, CNKI, VIP, and CBM) from inception through September 27, 2025. Cross-sectional studies evaluating peripheral blood protein levels of HIF-1α and VEGF in T2DM patients versus healthy controls were included. Data synthesis was performed using standardized mean differences (SMD) with 95% confidence intervals (CI). Heterogeneity and stability of the results were assessed via subgroup analysis, sensitivity analysis, and Egger’s test.

**Results:**

Thirty studies were included, comprising 6,400 T2DM patients and 2,994 healthy controls. Meta-analysis revealed significantly elevated peripheral blood levels of both HIF-1α and VEGF in T2DM patients compared to healthy controls. Subgroup analyses confirmed the robustness of these trends across all stratified populations. Sensitivity analyses demonstrated that the findings were robust, and Egger’s test indicated no evidence of significant publication bias.

**Conclusions:**

The distinct expression profiles of elevated HIF-1α and VEGF underscore their potential involvement in the pathophysiology of T2DM. Our meta-analysis confirms that T2DM is consistently associated with elevated circulating levels of both HIF-1α and VEGF. While these findings underscore the involvement of this molecular axis in T2DM pathophysiology, they do not support their current use as clinical biomarkers or therapeutic targets. The cross-sectional design of the included studies precludes causal inference. Future prospective and mechanistic research is essential to determine their potential utility.

**Systematic review registration:**

https://www.crd.york.ac.uk/PROSPERO/, identifier CRD420251164744

## 1 Introduction

The global prevalence of T2DM is rising steadily and is projected to affect 783 million individuals by 2045. Major complications include cardiovascular disease, diabetic nephropathy (DN), retinopathy (DR), peripheral neuropathy (DPN), and non-alcoholic fatty liver disease (NAFLD). Notably, patients with diabetes face a two- to four-fold increased risk of cardiovascular disease compared to the general population, with higher susceptibility observed in women and those with early-onset disease (1–3). Global healthcare expenditures for diabetes amounted to $966 billion in 2021 and are projected to exceed $1,054 billion in 2045, with the burden being heavier, especially in areas with limited healthcare resources, and posing an increasingly severe economic burden on the global public health system (1). While early detection of T2DM is essential for mitigating complications and reducing healthcare costs, the current physiological indicators for identifying early-stage disease remain inadequate (4). Beyond insulin resistance and β-cell dysfunction, which are core pathophysiological mechanisms of T2DM, hypoxic stress has emerged as an important contributing factor in recent years (5, 6). Hypoxia and inflammation are intertwined processes that together influence insulin sensitivity and beta-cell function (7).

In recent years, the role of multiple molecular regulators in T2DM has become increasingly prominent. HIF-1 is a heterodimeric transcription factor composed of an oxygen-labile alpha subunit (HIF-1α) and a constitutively expressed beta subunit (HIF-1β) (8). Under normoxic conditions, HIF-1α is hydroxylated and targeted for proteasomal degradation (8). Under hypoxic conditions, HIF-1α stabilizes, translocates to the nucleus, and dimerizes with HIF-1β to form active HIF-1, which then binds to hypoxia-response elements in target gene promoters (8). The transcriptional activity of HIF-1 is also modulated by cofactors such as NF-κB and HMGA1, both master regulators of inflammatory response (9–11). Clinical studies have reported that circulating HIF-1α levels are elevated in T2DM patients and correlate with disease progression and complications (12–14). Several studies have confirmed that peripheral blood HIF-1α levels in T2DM patients are significantly higher than those in the healthy population, and are positively correlated with blood glucose, HbA1c, insulin resistance, and other indicators (12, 13). In patients with DR, DN, atherosclerosis, and other complications, HIF-1α levels were further elevated, suggesting that it can be used as a potential biomarker for disease progression and complication risk (13, 14). At the molecular level, HIF-1α participates in multiple biological processes including metabolic regulation, inflammation, oxidative stress, angiogenesis, and organ damage (15–17). HIF-1α is an upstream transcription factor of VEGF. Through upregulation of VEGF, HIF-1α promotes angiogenesis and inflammatory responses that are implicated in the development of diabetic microvascular complications (18, 19). This HIF-1α/VEGF axis is a well-established pathway through which hypoxia drives pathological angiogenesis in diabetic complications such as retinopathy and nephropathy (20–22).

Although a considerable number of studies have examined changes in the expression of HIF-1α and VEGF in the peripheral blood of patients with T2DM, and most of them have reported directionally consistent trends, the evidence remains fragmented and lacks systematic integration. Individual studies often have limited sample sizes, varying characteristics of the study population (e.g., comorbidity status, disease duration), or different assays and sample types, resulting in a lack of precision in the estimation of their effects, making it difficult to form uniform conclusions that are sufficiently convincing. Therefore, there is an urgent need for a quantitative synthesis approach to critically assess and merge the available evidence to produce more robust and reliable summary estimates that can clarify the overall expression patterns of these molecular markers in patients with T2DM.

For these reasons, this study aimed to critically evaluate and quantitatively synthesize the available evidence by means of systematic review and meta-analysis, to elucidate the differences in peripheral blood expression levels of HIF-1α and VEGF between patients with T2DM and healthy populations. Through rigorous synthesis of evidence and exploration of heterogeneity, it is expected to provide higher-level evidence to clarify the role of these molecular mechanisms in the pathophysiological process of T2DM and may provide observational clues for further mechanistic investigations, but causal inference and clinical utility remain to be established.

## 2 Methods

The protocol for this systematic evaluation and meta-analysis is registered with PROSPERO (CRD420251164744, **Supplementary Material 1**). This study followed the PRISMA (Preferred Reporting Items for Systematic Evaluation and Meta-Analysis) 2020 guidelines. (**Supplementary Material 2**).

### 2.1 Retrieval strategy

We systematically searched electronic databases, including PubMed, Embase, the Cochrane Library, Web of Science, Wanfang, CNKI, VIP, and SinoMed from database inception through September 27, 2025. Search terms included: Search terms included: diabetes mellitus type 2, type II diabetes mellitus, hypoxia inducible factor 1, alpha subunit, hypoxia inducible factor 1alpha and HIF-1α. Both subject headings (MeSH terms or Emtree terms) and free-text words were combined and adapted for each electronic database. The detailed search strategies are provided in **Supplementary Material 3**.

### 2.2 Inclusion criteria

(1) Study population: adult patients (aged ≥18 years) diagnosed with T2DM, regardless of gender. Eligible studies were required to concurrently recruit a healthy control group comprising individuals without diabetes or other major systemic diseases. (2) Complication status: studies enrolling T2DM patients with or without diabetic complications were both eligible. Subgroup analyses were pre-specified to stratify by complication status and type. (3) Exposure factors: the exposure of interest was the difference in peripheral blood concentrations of HIF-1α, or VEGF (measured via ELISA) between the T2DM and control groups. (4) Primary outcome indicator: peripheral blood HIF-1α; secondary outcome indicator: peripheral blood VEGF expression level. (5) Study type: inclusion of observational studies, primary cross-sectional studies, or case-control studies.

### 2.3 Exclusion criteria

(1) Literature reviews, meta-analyses, conference abstracts, publications, and research; (2) Studies evaluated non-T2DM populations; (3) Studies failing to measure circulating HIF-1α or VEGF levels; (4) Literature that duplicated publications or had overlapping data (selecting for a larger sample size or higher quality); (5) Studies in which there was no established single-arm diabetes studies with inappropriate healthy control groups (e.g., healthy controls with other diseases); (6) literature of very low quality with unavailable data; (7) literature where the required data could not be obtained and contacting the authors was not fruitful; and (8) literature that was not in Chinese or English.

### 2.4 Literature screening and data extraction

Two researchers managed the literature independently using EndNote 20 software. After de-duplication, titles, abstracts, and full texts were read to screen the literature for meeting the inclusion criteria and to cross-check the literature. In case of disagreement, the decision was made through joint deliberation by a third researcher. Data from the literature were extracted using a pre-formulated Excel spreadsheet, and the information extracted included: first author, country, sample size, age, duration of disease, sex ratio, type of study, sample source, sample testing method, HIF-1α expression values, and VEGF expression values. If data were reported as median and range or interquartile range, we estimated the mean and standard deviation using the method proposed by Hozo et al. (23). If unit conversions were needed (e.g., ng/mL to pg/mL), we applied standard conversion factors. For studies with overlapping populations from the same research group, we included the one with the larger sample size or more complete data. Missing data were not imputed; we contacted corresponding authors for missing values when necessary.

### 2.5 Literature quality assessment

The quality of the included studies was evaluated independently by 2 investigators using the cross-sectional study evaluation criteria recommended by the Agency for Healthcare Research and Quality (AHRQ). The criteria consisted of 11 items, each of which could be answered “yes,” “no,” or “unclear.” The quality of the study was evaluated according to the score: 0–3 as low quality, 4–7 as moderate quality, and 8–11 as high quality. Disagreements were resolved through discussion or adjudication by a third researcher.

### 2.6 Certainty of evidence assessment

The certainty of evidence for each pooled outcome was independently evaluated by two investigators using the Grading of Recommendations, Assessment, Development and Evaluations (GRADE) framework. The assessment was structured across five standard domains including risk of bias, inconsistency, indirectness, imprecision, and publication bias. Based on the evaluation of these domains, the overall certainty of evidence for each molecular marker was classified into one of four distinct levels: high, moderate, low, or very low. Any disagreements during the grading process were resolved via mutual consensus or through consultation with a third reviewer.

### 2.7 Statistical analysis

The meta package of R 4.5.1 software was applied for statistical analysis. In this study, the outcome indicators were all continuous variables, and the Standardized Mean Difference (SMD) was used to synthesize the effect values and calculate the 95% confidence interval (CI). Differences between groups were considered statistically significant if the 95% CI did not contain zero. Heterogeneity between studies was measured using *I*² values; if *I*² ≤ 50%, it indicated low heterogeneity between studies and was analyzed using a fixed-effects model. If *I*² > 50%, it indicates high heterogeneity between studies and was analyzed using a random effects model. When significant heterogeneity existed, it was addressed by preplanned subgroup analyses (by sample type, region, presence of comorbidities, and severity of DN) to explore potential factors contributing to heterogeneity, and sensitivity analyses were used to assess the robustness of the results. The included literature was tested for publication bias by drawing funnel plots, and Egger’s test was used to assess publication bias. The test level *α* = 0.05.

## 3 Results

### 3.1 Literature screening and inclusion criteria

**Figure 1:**
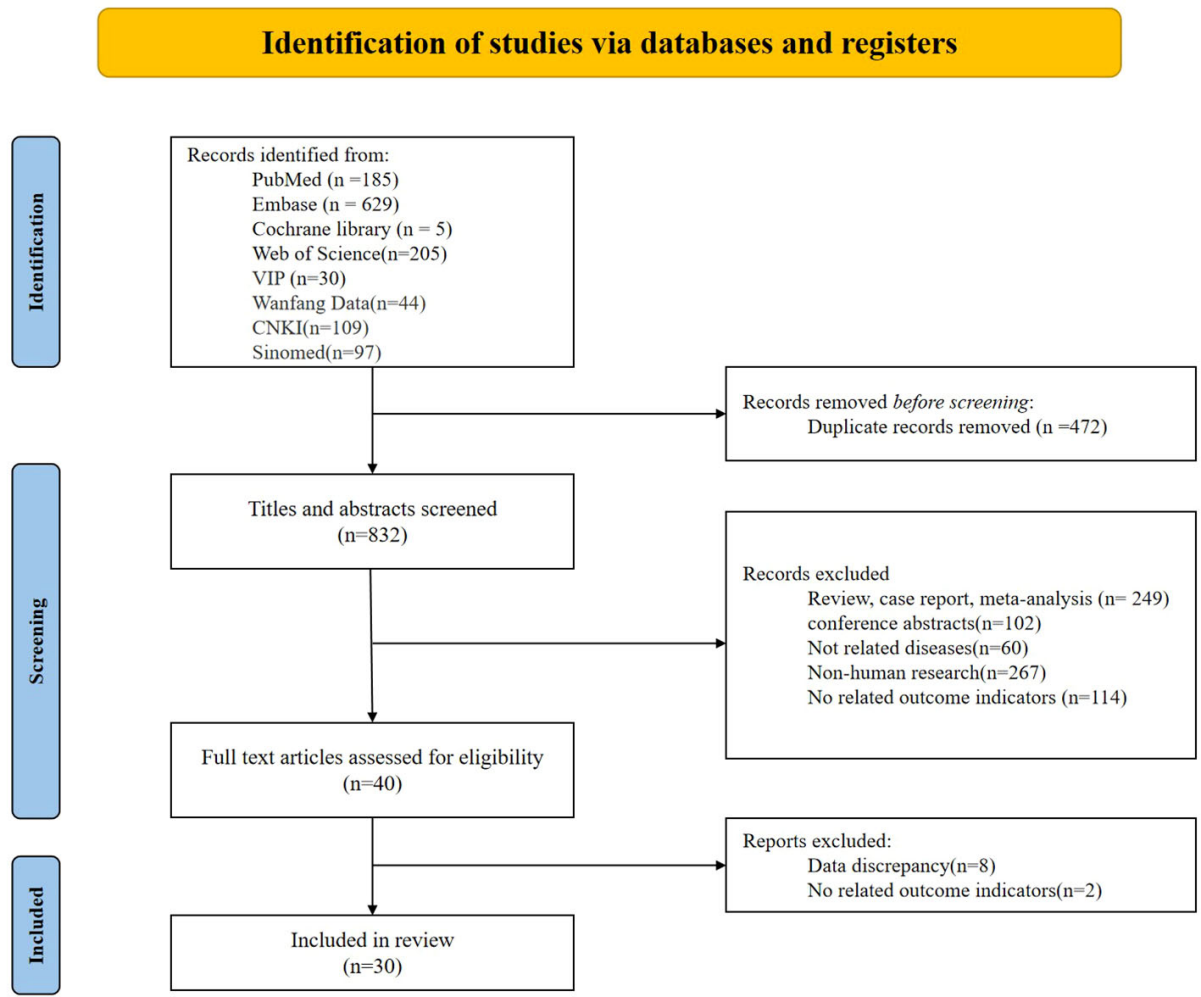
Literature screening flowchart.

One thousand three hundred and four relevant literature were obtained through database search, and 30 cross-sectional studies were finally included after de-emphasis, initial screening, and full-text re-screening assessment (**Figure 1**). The included studies involved 6,400 patients with T2DM and 2,994 healthy controls, all of which compared peripheral blood HIF-1α levels in patients with T2DM (test group) with those in the healthy population (control group), and the main characteristics are shown in **Table 1**. The studies were mainly from China (n = 27), which covers North and South China, and a few studies were from Italy, India, and Egypt (n = 3). All 30 studies reported peripheral blood HIF-1α levels, of which 14 also reported VEGF levels. The experimental group was comprised of T2DM patients, mostly with comorbidities such as DN, DR, and cervical or lower limb vasculopathy, while the control group consisted of healthy people with no related diseases. The age of the subjects was concentrated in the middle age stage, and the gender ratio was roughly balanced. The two groups were matched for age and sex in most studies. Peripheral blood HIF-1α expression levels were all measured by ELISA, and sample types were predominantly serum (n = 26) and partly plasma (n = 4). The quality of the included studies was assessed using the AHRQ checklist. Scores ranged from 7 to 10, with 28 of 30 studies scoring ≥ 8, indicating generally high methodological quality. Two studies scored 7 and were rated as moderate quality. The most frequently unmet items were consecutive enrollment, confounding control, and follow-up, with the latter being uniformly unmet due to the cross-sectional design. All studies satisfied items regarding the source of information, quality assurance, patient exclusions, missing data handling, and response rates. Sensitivity analysis excluding the two moderate-quality studies did not materially change the pooled effect estimates. A traffic-light plot summarizing the AHRQ assessment is provided in **Supplementary Material 4**.

**Table 1:** Basic characteristics of included studies.

General information					Demographic information of population				Layered indicators				Experimental									Control								Outcome	Quality score
First author	Publication year	Country	Region	Study design	Age	Sample size	Gender (M/F)		Method	Sample type	Matching (age/gender)	With or without complications	Age	Sample size	Gender (M/F)		Body mass index (BMI)	Duration (year)	HbA1c (%)	HIF-1α level		Age	Sample size	Gender (M/F)		BMI	HbA1c (%)	HIF-1α level			
Shao (24)	2016	China	North China	cross-sectional study	54.41	719	363	356	ELISA	serum	yes	with DN	55.00	508	255	253	25.54	8.72	7.35	29.72	ng/L	53.00	211	108	103	25.20	5.40	17.00	ng/L	HIF-1α	9
Zhou (25)	2017	China	South China	cross-sectional study	51.55	480	273	207	ELISA	serum	yes	with DN	51.28	240	141	99	25.37	/	/	30.68	pg/mL	51.81	240	132	108	25.45	/	15.30	pg/mL	HIF-1α	9
Shao (26)	2016	China	North China	cross-sectional study	53.51	671	320	351	ELISA	serum	yes	with DN	53.30	479	225	254	25.70	8.53	8.85	29.25	pg/mL	54.03	192	95	97	25.10	5.36	16.84	pg/mL	HIF-1α, VEGF	9
Shao (27)	2016	China	North China	cross-sectional study	54.16	616	317	299	ELISA	serum	yes	with DN	54.56	436	223	213	25.58	8.74	8.77	29.08	pg/mL	53.18	180	94	86	25.27	5.36	16.77	pg/mL	HIF-1α	9
E (28)	2012	China	South China	cross-sectional study	56.10	80	46	34	ELISA	serum	yes	with neck angiopathy	56.57	56	31	25	25.25	/	10.12	1.29	ng/mL	55.00	24	15	9	23.06	5.60	0.37	ng/mL	HIF-1α	9
Zhang (29)	2016	China	South China	cross-sectional study	54.34	78	40	38	ELISA	serum	no	with lower limb angiopathy	54.50	53	27	26	25.21	/	9.93	1.22	ng/mL	54	25	13	12.00	24.13	5.45	0.28	ng/mL	HIF-1α	9
Sun (30)	2024	China	South China	cross-sectional study	62.36	300	182	118	ELISA	serum	yes	with DN	62.69	200	122	78	/	/	7.67	0.77	ng/mL	61.70	100	60	40	/	4.54	0.53	ng/mL	HIF-1α	9
Han (31)	2023	China	North China	cross-sectional study	51.52	180	83	97	ELISA	serum	yes	with DN	51.25	90	40	50	26.42	/	11.85	30.72	pg/mL	51.78	90	43	47	26.50	5.08	12.34	pg/mL	HIF-1α	9
Jing (32)	2020	China	North China	cross-sectional study	52.38	162	91	71	ELISA	serum	yes	without complications	51.97	82	49	33	23.06	/	/	456.37	pg/mL	52.80	80	42	38	24.16	/	152.37	pg/mL	HIF-1α, VEGF	9
Ha (33)	2021	China	North China	cross-sectional study	53.36	94	47	47	ELISA	serum	no	without complications	/	54	/	/	/	/	/	4.40	ng/mL	/	40	/	/	/	/	4.44	ng/mL	HIF-1α, VEGF	7
Sun (34)	2021	China	North China	cross-sectional study	71.80	204	97	107	ELISA	serum	yes	with neck angiopathy	72.32	138	66	72	/	10.71	/	6.92	ng/L	70.71	66	31	35	/	/	0.37	ng/L	HIF-1α	8
Li (35)	2016	China	North China	cross-sectional study	55.43	122	66	56	ELISA	plasma	yes	with DN	54.50	92	50	42	/	/	6.14	211.21	pg/L	58.30	30	16	14	/	/	30.60	pg/L	HIF-1α, VEGF	7
Shan (36)	2010	China	South China	cross-sectional study	54.53	128	68	6	ELISA	serum	yes	with DN	55.00	98	52	56	23.17	7.80	10.90	97.71	pg/L	53.00	30	16	14	23.25	5.48	52.17	pg/L	HIF-1α, VEGF	9
Li (37)	2024	China	North China	cross-sectional study	67.01	300	215	85	ELISA	serum	yes	with lower limb angiopathy	66.70	200	145	55	24.61	16.21	7.70	314.79	pg/mL	67.65	100	70	30	24.03	4.87	70.59	pg/mL	HIF-1α	9
Shen (38)	2021	China	South China	cross-sectional study	/	60	/	/	ELISA	plasma	no	with cardiopathy	/	40	/	/	/	/	7.51	2.33	mg/mL	/	20	/	/	/	4.83	0.72	mg/mL	HIF-1α	9
Liu (39)	2013	China	South China	cross-sectional study	56.25	178	99	79	ELISA	serum	yes	with DN	55.64	132	74	58	/	4.89	9.86	252.43	ng/L	58.00	46	25	21	/	5.21	187.00	ng/L	HIF-1α, VEGF	9
Song (40)	2016	China	North China	cross-sectional study	55.80	99	53	46	ELISA	plasma	yes	with DN	55.10	76	41	35	23.19	7.15	6.16	200.77	pg/L	58.10	23	12	11	21.70	/	32.90	pg/L	HIF-1α, VEGF	9
Tian (41)	2020	China	North China	cross-sectional study	44.93	259	149	110	ELISA	serum	yes	with DR	44.55	195	115	80	23.03	/	/	26.71	pg/mL	46.10	64	34	30	22.80	/	23.80	pg/mL	HIF-1α, VEGF	9
Xiong (42)	2019	China	South China	cross-sectional study	45.35	400	215	185	ELISA	serum	yes	with DN/DR/lower limb angiopathy	44.50	200	110	90	/	/	8.21	612.25	pg/mL	46.20	200	105	95	/	5.81	166.45	pg/mL	HIF-1α	9
Xu (43)	2015	China	South China	cross-sectional study	56.33	240	132	108	ELISA	serum	yes	with neck angiopathy	56.50	160	87	73	26.00	/	10.10	1.25	ng/mL	56.00	80	45	35	25.00	5.60	0.39	ng/mL	HIF-1α	9
Zhang (44)	2023	China	North China	cross-sectional study	57.70	160	92	68	ELISA	serum	yes	with neck angiopathy	57.51	110	62	48	23.78	5.04	9.02	33.79	ng/mL	58.14	50	30	20	22.89	5.92	21.82	ng/mL	HIF-1α	9
Zhu (45)	2017	China	South China	cross-sectional study	/	690	/	/	ELISA	serum	yes	with DN	/	490	/	/	/	7.96	8.75	29.24	pg/mL	/	200	/	/	/	5.32	15.92	pg/mL	HIF-1α, VEGF	9
Bian (46)	2021	China	North China	cross-sectional study	63.84	413	204	209	ELISA	serum	yes	with DN	63.89	313	156	157	25.13	5.03	9.10	33.54	ng/L	63.67	100	48	52	24.66	5.45	21.56	ng/L	HIF-1α	9
Del Cuore (47)	2023	Italy	Not China	cross-sectional study	67.48	110	76	34	ELISA	serum	yes	with lower limb angiopathy	67.58	90	64	26	28.76	/	7.90	37.21	ng/mL	67.05	20	12	8	27.11	5.89	33.85	ng/mL	HIF-1α, VEGF	10
Gaonkar (48)	2020	India	Not China	cross-sectional study	59.34	518	/	/	ELISA	plasma	yes	with DR	59.56	370	/	/	27.20	6.78	8.80	1268.90	pg/mL	58.80	148	/	/	24.10	4.90	674.25	pg/mL	HIF-1α	9
Hu (49)	2017	China	South China	cross-sectional study	60.32	86	/	/	ELISA	serum	no	Unclear	60.84	60	/	/	/	/	/	2421.07	pg/mL	59.14	26	/	/	/	/	761.33	pg/mL	HIF-1α, VEGF	9
Ren (50)	2018	China	North China	cross-sectional study	55.55	563	292	271	ELISA	serum	yes	with DN	55.30	413	214	199	25.89	/	9.31	30.13	ng/L	56.23	150	78	72	25.86	5.06	17.55	ng/L	HIF-1α, VEGF	9
Sayed (51)	2016	Egypt	Not China	cross-sectional study	53.41	170	69	101	ELISA	serum	yes	with DR	53.00	100	39	61	27.15	11.00	9.90	169.10	pg/mL	54.00	70	30	40	25.06	5.90	108.70	pg/mL	HIF-1α	8
Shao (52)	2016	China	North China	cross-sectional study	49.45	726	366	360	ELISA	serum	yes	with DN	49.96	502	256	246	24.77	9.38	8.65	28.70	pg/mL	48.32	224	110	114	24.39	5.24	16.78	pg/mL	HIF-1α, VEGF	10
Shao (53)	2017	China	North China	cross-sectional study	49.77	588	298	290	ELISA	serum	yes	with DN	50.27	423	211	212	24.93	9.84	8.69	28.64	ng/L	48.50	165	87	78	24.50	5.20	16.60	ng/L	HIF-1α, VEGF	9

Slashes (/) indicate that the specific clinical characteristics (e.g., BMI, T2DM duration or gender) were not reported in the original study or could not be derived from the provided data.

### 3.2 Meta-analysis results

#### 3.2.1 Outcome indicator

##### 3.2.1.1 Primary outcome measure

###### 3.2.1.1.1 HIF-1α

**Figure 2:**
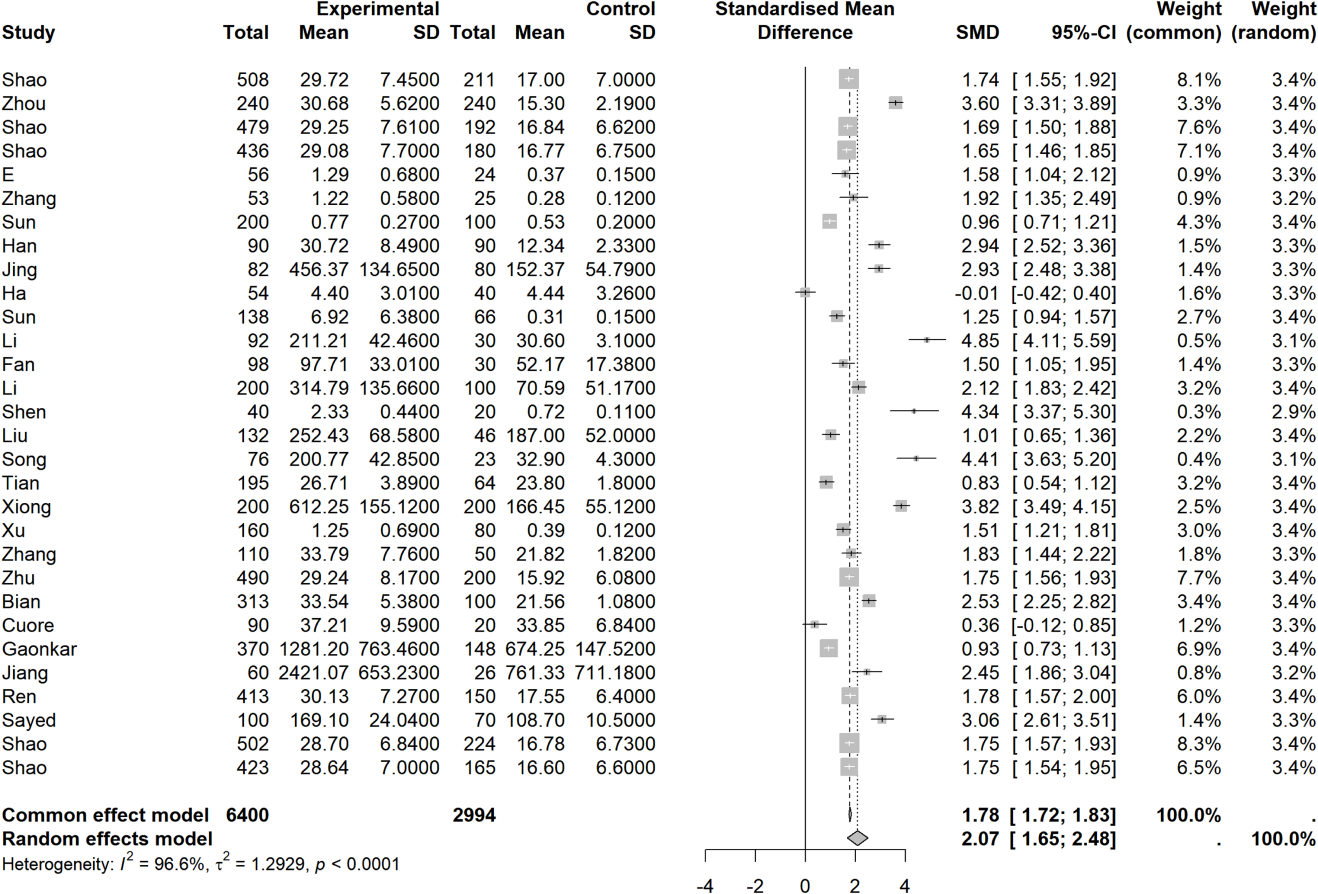
HIF-1α forest plot.

A total of 30 papers reported peripheral blood HIF-1α expression, and meta-analysis showed that the SMD (SMD = 2.07; 95% CI: 1.65 to 2.48, *P* < 0.0001) of HIF-1α combined was positive in the patients in the test and control groups, which indicates that T2DM patients exhibited significantly elevated levels of peripheral HIF-1α compared to healthy controls. These findings suggest that high HIF-1α expression is consistently associated with the development of T2DM across the included studies, potentially playing a role in its pathophysiological progression. However, inter-study heterogeneity was relatively high (*I*² = 96.6%, *P* < 0.0001), and therefore a random effects model was used for the analysis (**Figure 2**).

##### 3.2.1.2 Secondary outcome measures

###### 3.2.1.2.1 VEGF

**Figure 3:**
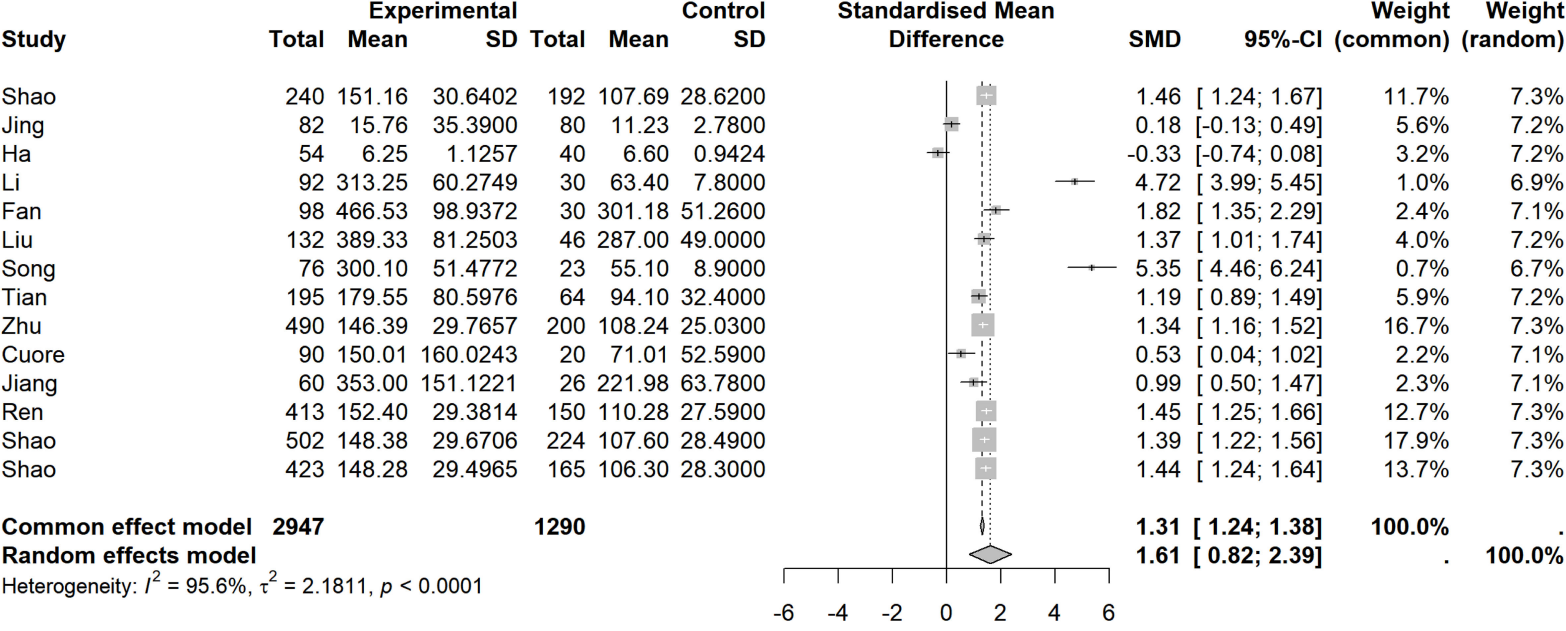
VEGF forest plot.

A total of 14 papers reported peripheral blood VEGF expression levels. Meta-analysis showed that VEGF levels were significantly higher in the T2DM group than in the control group (SMD = 1.61; 95% CI: 0.82 to 2.39, *P* < 0.0001). Heterogeneity among the 14 included studies was high (*I²* = 95.6%, *P* < 0.0001), so a random-effects model was used (**Figure 3**).

#### 3.2.2 Subgroup analysis

Given that the outcome metrics (HIF-1α and VEGF) were all significantly heterogeneous (*I²* > 95%), the present study performed systematic subgroup analyses based on sample type (serum vs. plasma), region (northern China vs. southern China vs. non-Chinese), and comorbidities status (no comorbidities vs. combined comorbidities), to explore the potential sources of heterogeneity. In particular, the T2DM population with comorbid DN was further subdivided into subgroups based on their disease severity to carefully assess the potential impact of DN severity on the expression levels of the above molecules.

HIF-1α: In all predefined subgroups, HIF-1α expression was significantly higher in T2DM patients than in healthy controls (*P* < 0.0001). In patients with DN, HIF-1α expression levels tended to increase with increasing disease severity: SMD = 3.22 (95% CI: 1.98 to 4.46) in the microalbuminuria group and SMD = 4.12 (95% CI: 2.76 to 5.48) in the macroalbuminuria group. Heterogeneity within the remaining subgroups remained high (*I*² > 95%), suggesting the presence of other unmeasured sources of heterogeneity (**Table 2**).

**Table 2:** Subgroup analysis results for HIF-1α.

Item	Subgroup	Number of studies included	Results of the heterogeneity test		Effects models	Meta-analysis results		
			I² (%)	P		SMD	95% CI	P
Region	North China	17	95.1	<0.0001	Random	2.09	[1.55; 2.62]	<0.0001
	South China	10	97.6	<0.0001		2.22	[1.48; 2.97]	
	Not China	3	97.6	<0.0001		2.07	[1.65; 2.48]	
Sample type	Serum	26	96.1	<0.0001	Random	1.86	[1.51; 2.20]	<0.0001
	Plasma	4	98.5	<0.0001		3.60	[1.78; 5.42]	
With or without complications	without complications	24	97.5	<0.0001	Random	2.07	[1.26; 2.88]	<0.0001
	with complications	24	97.4	<0.0001		2.50	[1.93; 3.07]	
Type of complications	with DN	13	98.1	<0.0001	Random	3.02	[2.06; 3.99]	<0.0001
	with neck angiopathy	3	0	0.9910		2.11	[1.84; 2.37]	
	with lower limb angiopathy	4	96.2	<0.0001		2.49	[1.03; 3.94]	
	with DR	3	95.7	<0.0001		1.96	[0.68; 3.23]	
Severity of DN	Microalbuminuria	12	97.3	<0.0001	Random	3.22	[1.98; 4.46]	<0.0001
	Macroalbuminuria	12	97.2	<0.0001		4.12	[2.76; 5.48]	

VEGF: Analysis of the subgroups consistently showed that VEGF expression was significantly higher in T2DM patients than in healthy controls (*P* < 0.0001). DN severity was positively correlated with VEGF expression levels, with a significantly higher effect size in the massive albuminuria group (SMD = 3.05) than in the microalbuminuria group (SMD = 1.54). Heterogeneity within subgroups remained high (*I*² > 95%), suggesting the presence of other unmeasured sources of heterogeneity (**Table 3**).

**Table 3:** Subgroup analysis results for VEGF.

Item	Subgroup	Number of studies included	Results of the heterogeneity test		Effects models	Meta-analysis results		
			I² (%)	P		SMD	95% CI	P
Region	North China	9	97.2	<0.0001	Random	1.84	[0.62; 3.06]	<0.0001
	South China	4	50.5	<0.0001		1.37	[1.13; 1.61]	
Sample type	Serum	12	91.6	<0.0001	Random	1.07	[0.73; 1.42]	<0.0001
	Plasma	2	13.0	0.2838		4.98	[4.37; 5.59]	
With or without complications	without complications	9	26.8	<0.0001	Common	0.92	[0.83; 1.01]	<0.0001
	with complications	9	97.4	<0.0001	Random	1.82	[1.52; 2.12]	
Severity of DN	Microalbuminuria	7	64.1	0.0103	Random	1.54	[1.36; 1.72]	<0.0001
	Macroalbuminuric	7	75.1	0.0005		3.05	[2.75; 3.35]	

#### 3.2.3 Regression analysis

**Figure 4:**
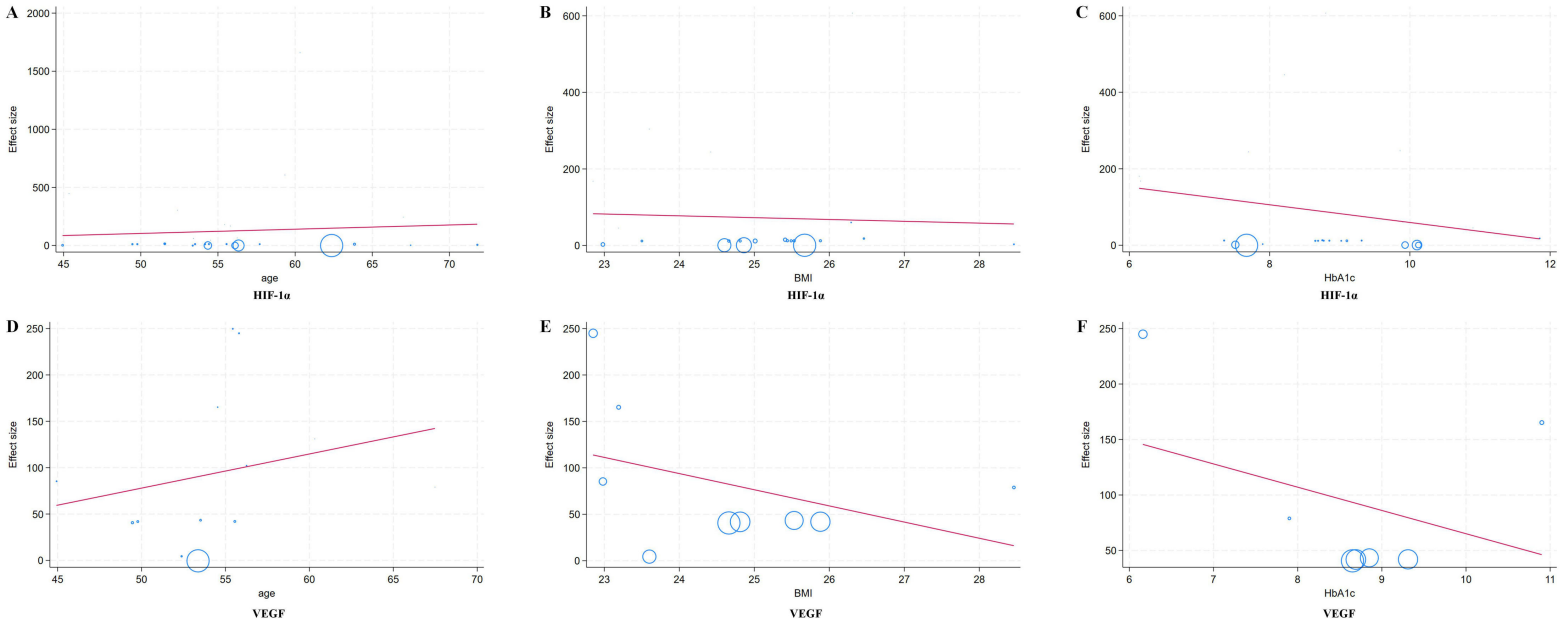
Regression analysis **(A)** the regression analysis of age and HIF-1α; **(B)** the regression analysis of BMI and HIF-1α; **(C)** the regression analysis of HbA1c and SIRT1; **(D)** the regression analysis of age and VEGF; **(E)** the regression analysis of BMI and VEGF; **(F)** the regression analysis of HbA1c and VEGF.

Due to the high inter-study heterogeneity, we performed meta-regression analyses using restricted maximum likelihood estimation (REML) to explore potential sources of heterogeneity. We examined the effects of age, BMI, and HbA1c on the observed effect sizes. Meta-regression showed that none of these covariates had a significant effect on HIF-1α or VEGF levels between T2DM patients and healthy controls (*P* > 0.05), suggesting that the high heterogeneity was primarily driven by individual-level differences within studies rather than by these aggregate study-level characteristics (**Figure 4**). In particular, HbA1c was not a significant source of heterogeneity, indicating that differences in glycemic control across studies did not explain the observed variability. Residual heterogeneity remained substantial, indicating that future studies should collect individual participant data to further explore potential confounding factors.

#### 3.2.4 Sensitivity analysis

**Figure 5:**
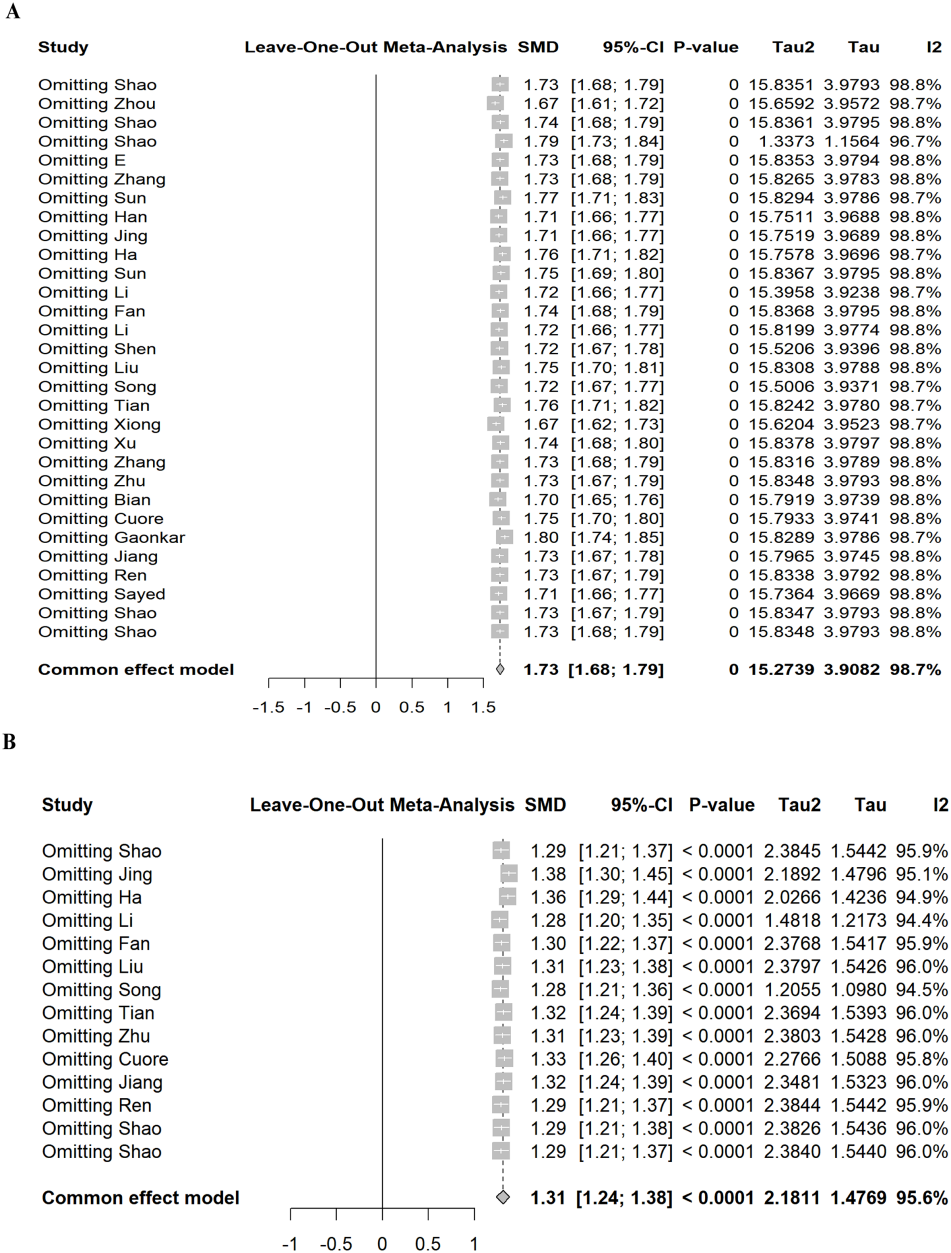
Sensitivity analysis **(A)** sensitivity analysis of HIF-1α; **(B)** sensitivity analysis of VEGF.

Due to some heterogeneity among the included studies, we conducted sensitivity analyses using the one-by-one exclusion method to assess the robustness of the combined results. The results of the analysis showed that the pooled effect sizes were not substantially altered after excluding each study in turn, and the direction of their associations remained consistent throughout. This suggests that the primary outcome of this study is more reliable and not overly dependent on any single study. The results are shown in **Figure 5**.

#### 3.2.5 Publication bias

**Figure 6:**
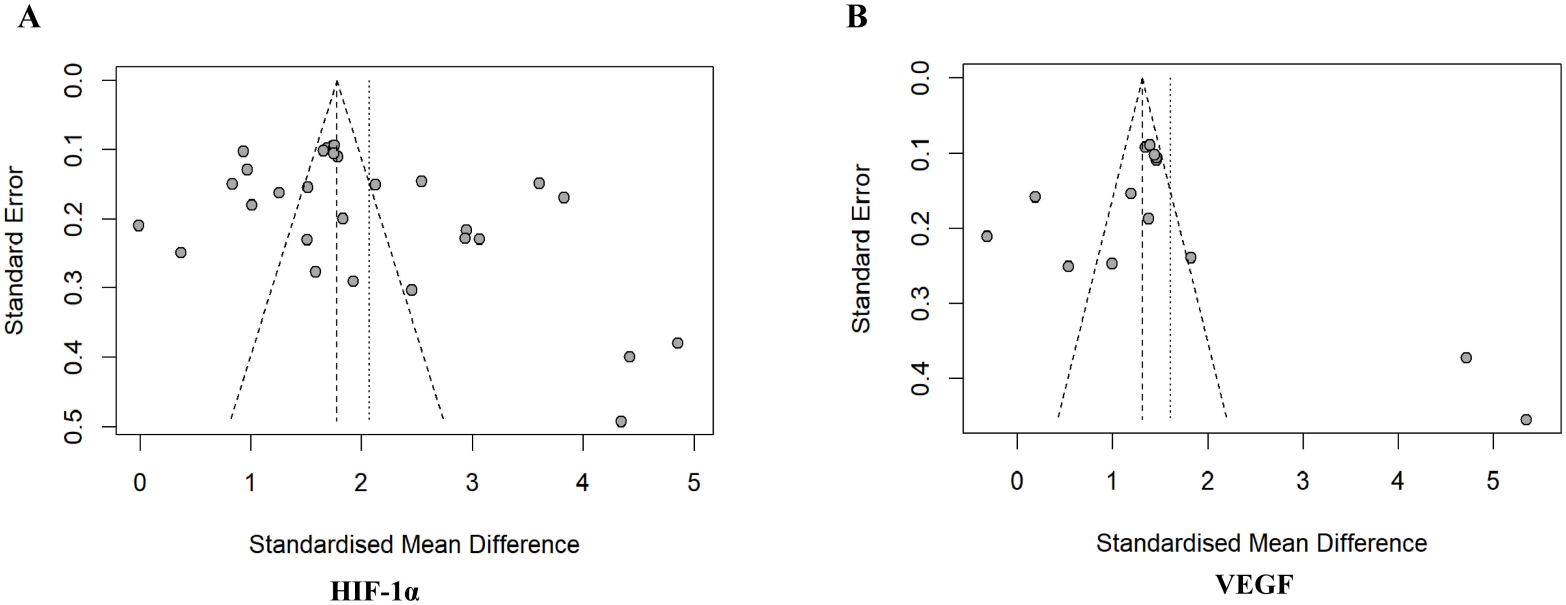
Funnel plot **(A)** funnel plot of HIF-1α; **(B)** funnel plot of VEGF.

To assess potential publication bias, the distribution symmetry of the included studies was visually assessed by funnel plots and quantitatively analyzed using Egger’s test (**Figure 6**). For HIF-1α and VEGF, the funnel plots were roughly symmetrical, and Egger’s test did not indicate significant publication bias (*P* = 0.07 and 0.49, respectively). However, these tests have limited power with the current number of studies, and the *P* value for HIF-1α (0.07) approached the significance threshold. The above results, together with the sensitivity analyses, support the overall reliability of the main findings, but the possibility of publication bias cannot be completely excluded.

#### 3.2.6 Certainty of evidence

The certainty of evidence for the primary and secondary outcomes was systematically graded. For the primary outcome, peripheral blood HIF-1α levels, the evidence body started at a low certainty baseline owing to the observational nature of the included cross-sectional studies. The certainty was further downgraded to very low due to severe unexplained statistical heterogeneity across trials and potential selection biases identified during quality assessment. Similarly, the certainty of evidence for circulating VEGF levels was adjudicated as very low, driven by extensive statistical inconsistency. The detailed GRADE evidence profiles and reasons for downgrading are comprehensively summarized in **Table 4**.

**Table 4:** Summary table of GRADE evidence.

Outcome	No. of studies (patients)	Risk of bias	Inconsistency	Indirectness	Imprecision	Publication bias	Certainty
HIF-1α	30 studies (9,394)	Serious ^a^	Very serious ^b^	Not serious	Not serious	Undetected	Very Low
VEGF	14 studies (5,635)	Serious ^a^	Very serious ^b^	Not serious	Not serious	Undetected	Very Low

^a^Downgraded by one level due to methodological limitations in primary cross-sectional studies, particularly regarding the lack of consecutive enrollment and insufficient control for confounding variables. ^b^Downgraded by two levels due to extremely high statistical heterogeneity ( $I^{2}>95\%$) that remained unresolved after comprehensive subgroup and meta-regression analyses.

## 4 Discussion

The main findings of the systematic evaluation and meta-analysis in this study were as follows: (1) Peripheral blood HIF-1α expression levels were significantly higher in T2DM patients than in healthy controls (SMD = 2.07; 95% CI: 1.65 to 2.48; *I²* = 96.6%), and VEGF expression was also significantly upregulated (SMD = 1.61; 95% CI: 0.82 to 2.39; *I²* = 95.6%); (2) Subgroup analyses reveal that the directional trends of these expression differences remain entirely consistent across all predefined strata, including various sample types, geographic distributions, complication statuses, and severities of diabetic nephropathy. However, the pooling of clinically diverse populations with distinct complications constitutes a major source of heterogeneity that requires cautious interpretation. (3) Sensitivity analysis confirmed that the combined results were robust and not overly dependent on any single study; (4) Publication bias assessment showed that no significant publication bias was detected in the HIF-1α or VEGF-related studies.

While the directional consistency of these markers supports their involvement in T2DM, the substantial clinical and statistical heterogeneity warrants careful interpretation. The inclusion of T2DM patients both with and without complications introduced clinical heterogeneity. However, pooling these populations allowed us to establish a comprehensive baseline for the HIF-1α/VEGF axis. This approach helps evaluate whether circulating levels reflect a systemic response to chronic hyperglycemia. Because T2DM is fundamentally a systemic metabolic disorder, localized tissue hypoxia and metabolic stress often precede clinical manifestations in specific organs. Therefore, combining these populations allows us to evaluate whether circulating biomarker levels reflect an overarching systemic response to chronic hyperglycemia. However, this broad pooling strategy inevitably limits the direct interpretability of the overall effect estimates, as the pathophysiological drivers of microvascular complications may differ from macrovascular conditions. Although our subgroup analyses stratified by complication status and type, the persistent high heterogeneity within subgroups implies that other unmeasured variables continue to influence circulating levels. For instance, medications commonly used in T2DM management, variations in disease duration, and differences in glycemic control were not uniformly adjusted for in the primary studies. Consequently, investigators should interpret the overall pooled estimates as a generalized systemic trend rather than a precise diagnostic metric for specific complication subtypes.

In T2DM, tissues generally face hypoxia, yet HIF-1α expression exhibits complex, tissue-specific bidirectional regulation. While HIF-1α can protect cells by adapting oxygen metabolism to hypoxic environments, its aberrant activation often exacerbates tissue damage and complications. This tissue hypoxia results from impaired vascular perfusion, metabolic abnormalities, and reduced hypoxic adaptation (54–57). For instance, high glucose increases HIF-1α and ADAM17 expression in renal mesangial cells, accelerating fibrosis, whereas it inhibits HIF-1α in proximal tubule cells (54–57). Conversely, chronic high glucose exposure can blunt the hypoxic stabilization of HIF-1α in proximal tubule cells (57). This blunting effect is fully reversed by PHD inhibition or VHL deficiency. Regarding diabetic retinopathy, HIF-1α plays a protective, anti-inflammatory role early on, despite its established detrimental role in driving angiogenesis during the proliferative phase (58). Similarly, in pancreatic β-cells, the role of HIF-1α remains contradictory; reduced expression is linked to impaired insulin release in some studies, while other research suggests its deletion improves β-cell function disrupted by VHL deficiency (59–61). HIF-1α directly binds to the hypoxia-responsive element in the VEGF gene promoter region, thereby upregulating VEGF transcription and promoting angiogenesis (20). In diabetic retinopathy, hypoxia-induced activation of the HIF-1α/VEGF pathway is a major driver of pathological neovascularization (21, 22). Importantly, elevated circulating HIF-1α levels reflect a systemic response to metabolic stress and do not necessarily parallel localized tissue expression (6). Consequently, early diabetic microcirculatory insufficiency activates the HIF-1α/VEGF pathway to improve oxygen supply, yet this response often becomes dysregulated, causing either insufficient activation or excessive, pathological angiogenesis (21, 22). Local cellular responses vary by hypoxic duration. Acute hypoxia induces adaptive HIF-1α stabilization to optimize oxygen utilization and maintain vascular integrity. Conversely, chronic hyperglycemia and prolonged hypoxia turn sustained HIF-1α upregulation into a pathological driver of cellular damage and tissue fibrosis (62). This pathological transition relies on molecular crosstalk (5). Metabolic stress triggers mitochondrial dysfunction and reactive oxygen species accumulation. This oxidative state stabilizes HIF-1α independently of oxygen tension, amplifying vascular inflammation (55). Persistent HIF-1α activation leads to excessive VEGF expression, which promotes the formation of immature and leaky microvessels that drive proliferative retinopathy and nephropathy (21).

Our findings are in line with the direction reported in most of the included studies, for example, the upregulation of HIF-1α was generally larger in studies targeting the Chinese population, which may reflect the influence of genetic background or environmental factors in specific populations. Compared with previous sporadic studies, the present analysis confirms the prevalence and stability of these molecular alterations in patients with T2DM through a larger sample size and more stringent inclusion criteria. Of particular note, subgroup analyses showed that differences in the expression of these molecules persisted regardless of whether patients had concomitant complications, suggesting that they may be early events in the core pathophysiologic processes of T2DM rather than merely secondary phenomena of complications. In patients with T2DM, elevated peripheral blood levels of HIF-1α usually suggest that tissues are in a state of functional hypoxia, and based on the multiple clinical and basic studies described above, we hypothesize that elevated HIF-1α may not be the primary driver of T2DM, but rather a biological indicator of chronic hypoxia and the body’s dysregulated response to long-term oxygen metabolism.

Despite the high methodological quality scores obtained via the AHRQ checklist, the overall certainty of evidence evaluated using the GRADE framework was determined to be very low for both molecular outcomes. Several critical limitations within the available evidence body warrant cautious interpretation. First, approximately 90% of the included studies were conducted in China, with only three studies originating from other countries. This significant geographic imbalance substantially restricts the generalizability of our findings to other ethnicities and healthcare systems, particularly given potential differences in genetic backgrounds, lifestyle factors, and clinical practice patterns. Second, all included studies were cross-sectional in design, which precludes any causal inference between biomarker levels and T2DM development or progression. Third, the very high statistical heterogeneity (I² > 95%) across outcomes remained largely unexplained even after subgroup and meta-regression analyses, indicating that unmeasured factors likely contribute. Fourth, differences in ELISA kits, specimen handling, and sample types (serum vs. plasma) may have introduced measurement variability. Fifth, most primary studies did not adjust for key confounders such as medication use, smoking, and hypertension. Although we included HbA1c in our meta-regression analyses and found no significant effect, we cannot exclude the possibility that other unmeasured variables, such as disease duration and medication types, may contribute to the observed heterogeneity. Sixth, due to limitations in raw data access, we were unable to perform more in-depth analyses for all potential influences. Finally, all primary literature included in this systematic review used a cross-sectional design, which inherently limits our ability to establish temporal or causal relationships. Although we utilized the rigorous AHRQ checklist to ensure high methodological quality among the selected studies, the inherent limitations of observational evidence mean the pooled estimates should be interpreted with caution. Future prospective longitudinal studies are needed to confirm these findings.

Based on the findings and limitations of this study, the following directions may be considered for future studies: first, more rigorous prospective cohort studies are needed to clarify the temporal relationship between changes in relevant molecular mechanisms and the risk of developing T2DM and their predictive value. Second, standardized sample processing and testing procedures should be established to improve the comparability of results between different studies. Third, given that molecules such as HIF-1α and VEGF are widely involved in the early pathological process of diabetes, they may not be ideal biomarkers for specific complications. Future studies should focus on examining its expression heterogeneity and spatiotemporal dynamic changes in different tissue microenvironments. Instead of treating it as a single qualitative marker, in-depth exploration and establishment of regression models combining multiple indicators or combining it with specific clinical phenotypes (e.g., microalbuminuria or fundus imaging features) are expected to screen out molecular signature profiles with high discriminatory power for specific complications, thus realizing more precise early risk warning and stratified management. Finally, further in-depth basic research should be conducted to reveal the specific signaling pathways of relevant molecules in the pathogenesis of T2DM and provide a theoretical basis for the development of novel therapeutic strategies. The advancement of these studies will deepen the systematic understanding of the molecular mechanisms of T2DM and its complications and provide new scientific pathways for early diagnosis and individualized treatment.

## 5 Conclusion

This systematic review and meta-analysis demonstrates that peripheral blood levels of HIF-1α and VEGF are significantly elevated in patients with T2DM. Subgroup analyses further confirm that these expression patterns remain consistent across different sample types, geographical regions, and comorbidity statuses. Furthermore, the upregulation of both HIF-1α and VEGF correlates positively with the severity of diabetic nephropathy. In summary, our meta-analysis demonstrates consistent associations between circulating HIF-1α and VEGF levels and T2DM across the available literature. However, due to the cross-sectional design of the included studies and the substantial unexplained heterogeneity, these findings should not be interpreted as supporting clinical monitoring or therapeutic targeting at this time. Instead, prospective longitudinal and mechanistic studies are required to clarify their predictive value and causal roles.

## Data Availability

The original contributions presented in the study are included in the article/**Supplementary Material**. Further inquiries can be directed to the corresponding author.
