# Supplementary material for: Peripheral blood HIF-1α levels in type 2 diabetes mellitus and their association with diabetic complications: a systematic review and meta-analysis
Source: Front Endocrinol (Lausanne). 2026 Jul 29;17:1879557. doi: 10.3389/fendo.2026.1879557 (PMC13461533; doi:10.3389/fendo.2026.1879557)
Supplement: Supplementary file 1 [file SupplementaryFile1.pdf]

NIHR | National Institute for Health and Care Research

PROSPERO
International prospective register of systematic reviews

Systematic review and meta-analysis of HIF-1αexpression differences in peripheral blood of patients with type 2 diabetes mellitus:a comparative analysis with healthy people

Yi Su, Rui Peng, Yunhui Chen

Citation
Yi Su, Rui Peng, Yunhui Chen. Systematic review and meta-analysis of HIF-1αexpression differences in peripheral blood of patients with type 2 diabetes mellitus:a comparative analysis with healthy people. PROSPERO 2025 CRD420251164744. Available from https://www.crd.york.ac.uk/PROSPERO/view/CRD420251164744.

REVIEW TITLE AND BASIC DETAILS

Review title

Systematic review and meta-analysis of HIF-1αexpression differences in peripheral blood of patients with type 2 diabetes mellitus:a comparative analysis with healthy people

Condition or domain being studied

Type 2 Diabetes Mellitus; Hypoxia

Rationale for the review

There are differences in a variety of inflammatory factors and metabolic indicators between diabetic patients and healthy people.Are there differences in hypoxia stress indicators? Hypoxia inducible factor- 1α(HIF-1a)as a sensor of cellular oxygen metabolism,its basic level in diabetic patients deserves attention.On the one hand,the theoretical mechanism:T2DM patients often have chronic hyperglycemia,insulin resistance,etc.,which lead to tissue microcirculation disorders and increased risk of local hypoxia.These factors may induce the expression of HIF-1ain peripheral blood to adapt to chronic hypoxic environment.However,some studies have found that hyperglycemia can accelerate the degradation of HIF-1a,resulting in "insufficient hypoxia response".Sporadic studies have reported the level of HIF-1ain the blood of patients with T2DM:some studies have pointed out that the expression of HIF-1ain patients with diabetes is lower than that in normal people,while other studies have reported that the level of HIF-1ain the serum of patients with diabetes is higher than that in healthy people.For example,HIF-1αin patients with

uncomplicated T2DM is significantly higher than that in normal control, and the incidence of diabetes complicated with complications is higher.The inconsistency of these results may be due to the differences in the course of disease and whether there are complications, which need to be explained by systematic review and meta-analysis.

Review objectives

How does the expression of HIF-1a in peripheral blood differ between patients with type 2 diabetes mellitus and healthy people?

Keywords

Type 2 diabetes mellitus; Hypoxia-inducible factor 1α; Healthy controls

Country

China

ELIGIBILITY CRITERIA

Population

Included

Adult patients diagnosed with type 2 diabetes mellitus,regardless of the duration.(as diagnosed using any recognised diagnostic criteria).During the same period of the study,healthy control group was

recruited,that is,individuals without diabetes and basically healthy(without major diseases).

Intervention(s) or exposure(s)

Included

Glucose

Comparator(s) or control(s)

This review does not have any comparators

Study design

Only nonrandomized study types will be included.

Context

The included study needs to be an observational study conducted in the hospital.The subjects are adults ⩾18 years old and diagnosed with type 2 diabetes.The same study needs to include healthy people as the control.This study must report the expression level of HIF-1α(detected in peripheral blood,plasma, or serum).The research location is not limited,but is limited to human research and does not include animals,intervention trials,conference summaries,or comments.

TIMELINE OF THE REVIEW

Date of first submission to PROSPERO

09 October 2025

Review timeline
Start date: 9 October 2025. End date: 31 May 2026.

Date of registration in PROSPERO
09 October 2025

AVAILABILITY OF FULL PROTOCOL

Availability of full protocol
A full protocol has not been written.

SEARCHING AND SCREENING

Search for unpublished studies
Only published studies will be sought.

Main bibliographic databases that will be searched
The main databases to be searched are *CENTRAL - Cochrane Central Register of Controlled Trials*, *CLIB - The Cochrane Library*, *Embase - Embase via Ovid*, *Embase.com*, *MEDLINE*, *PubMed*, *SCI - Science Citation Index* and *SSCI - Social Science Citation Index*.

Search language restrictions
The review will only include studies published in English and Chinese.

Search date restrictions
There are no search date restrictions.

Other methods of identifying studies
No other methods will be used.

Link to search strategy
A full search strategy is available in the full protocol as described in the *Availability of full protocol* section

Selection process
Studies will be screened independently by at least two people (or person/machine combination) with a process to resolve differences.

Other relevant information about searching and screening
None

DATA COLLECTION PROCESS

Data extraction from published articles and reports
Data will be extracted independently by at least two people (or person/machine combination) with a process to resolve differences.

Authors will be asked to provide any required data not available in published reports.

Study risk of bias or quality assessment

Risk of bias will be assessed using: *Newcastle-Ottawa*

Data will be assessed independently by at least two people (or person/machine combination) with a process to resolve differences.

Additional information will be sought from study investigators if required information is unclear or unavailable in the study publications/reports.

Reporting bias assessment
Risk of bias due to missing results will be assessed

Certainty assessment
Calculate the effect size(e.g.,relative risk-RR,odds ratio-OR,mean difference-MD)and its 95%confidence interval(95%Cl)foreach included study.Narrower Cls indicate higher certainty in the effect size estimate.If the Cl includes the null value(e.g.,RR or OR=1,MD=0),it shows statistical non-support for the effect,reflecting evidence uncertainty.

OUTCOMES TO BE ANALYSED

Main outcomes
Expression level of HIF-1ain peripheral blood

Additional outcomes
There are no additional outcomes.

PLANNED DATA SYNTHESIS

Strategy for data synthesis
The random effect dersimonian Laird model(Hartung Knapp correction)was used to combine the standardized mean difference(SMD)or risk ratio(RR)to quantify the correlation between HIF-1aandβ cell function;The heterogeneity was evaluated by I^2 and Q tests,and the influence of sample source,course of disease,BMI and other confounding factors was explored by subgroup analysis;Further sensitivity analysis was performed by eliminating articles one by one,and funnel egger test was used to determine whether the included studies had publication bias;All analyses were completed in R.4.5.1,and Stata 18and the results strictly followed the PRISMA 2020 reporting specification.

CURRENT REVIEW STAGE

Stage of the review at this submission

Review stage	Started	Completed
Pilot work	✓	
Formal searching/study identification	✓	
Screening search results against inclusion criteria		
Data extraction or receipt of IPD		
Risk of bias/quality assessment		

Review stage	Started	Completed
Data synthesis		

Review status
The review is currently planned or ongoing.

Publication of review results
Results of the review will be published.

REVIEW AFFILIATION, FUNDING AND PEER REVIEW

Review team members
Ms Yi Su (review guarantor and contact) ORCID: 0009-0007-6131-6542. Chengdu University of Traditional Chinese Medicine. China.

No conflict of interest declared.

Rui Peng. Chengdu University of Traditional Chinese Medicine. China.

No conflict of interest declared.

Yunhui Chen. Chengdu University of Traditional Chinese Medicine. China.

No conflict of interest declared.

Named contact
Ms Yi Su (1426965520@qq.com). ORCID: 0009-0007-6131-6542. Chengdu University of Traditional Chinese Medicine. China.

Review affiliation
Chengdu University of Traditional Chinese Medicine

Funding source
Review has no specific/external funding but is supported by guarantor/review team (non-commercial) institutions.

Peer review
There has been no peer review of this planned review.

ADDITIONAL INFORMATION

Review conflict of interest
Declared individual interests are recorded under team member details.. No additional interests are recorded for this review.

Medical Subject Headings
Diabetes Mellitus, Type 2; Hypoxia-Inducible Factor 1, alpha Subunit

SIMILAR REVIEWS

Check for similar records already in PROSPERO

PROSPERO identified a number of existing PROSPERO records that were similar to this one (last check made on 9 October 2025). These are shown below along with the reasons given by that the review team for the reviews being different and/or proceeding.

- Association between hypoxia-inducible factor-1α (HIF-1α) expression and pancreatic β-cell dysfunction in patients with type 2 diabetes mellitus: a systematic review and meta-analysis [published 3 August 2025] [CRD420251118501]. The review was judged **not to be similar**
- Effect of Liraglutide on blood pressure in patients with type 2 diabetes mellitus: a systematic review and meta-analysis [published 30 April 2021] [CRD42021244256]. The review was judged **not to be similar**
- Polymorphic variants and risk of diabetic peripheral neuropathy in patients with type 2 diabetes mellitus: systematic review and meta-analysis [published 12 February 2024] [CRD42024505256]. The review was judged **not to be similar**

PROSPERO version history

- Version 1.0, published 09 Oct 2025

Disclaimer

The content of this record displays the information provided by the review team. PROSPERO does not peer review registration records or endorse their content.

PROSPERO accepts and posts the information provided in good faith; responsibility for record content rests with the review team. The guarantor for this record has affirmed that the information provided is truthful and that they understand that deliberate provision of inaccurate information may be construed as scientific misconduct.

PROSPERO does not accept any liability for the content provided in this record or for its use. Readers use the information provided in this record at their own risk.

Any enquiries about the record should be referred to the named review contact
