# Supplementary material for: Peripheral blood HIF-1α levels in type 2 diabetes mellitus and their association with diabetic complications: a systematic review and meta-analysis
Source: Front Endocrinol (Lausanne). 2026 Jul 29;17:1879557. doi: 10.3389/fendo.2026.1879557 (PMC13461533; doi:10.3389/fendo.2026.1879557)
Supplement: Supplementary file 3 [file SupplementaryFile3.pdf]

Pubmed

Search	Query	Results	Date
#1	"diabetes mellitus, type 2"[MeSH Terms]	192996	2025/09/27
#2	"type II diabetes mellitus"[Title/Abstract] OR "type II diabetes"[Title/Abstract] OR "type 2 diabetes*"[Title/Abstract] OR "TIIDM"[Title/Abstract] OR "T2DM"[Title/Abstract] OR "Stable Diabetes Mellitus"[Title/Abstract] OR "noninsulin dependent diabetes*"[Title/Abstract] OR "non insulin dependent diabetes*"[Title/Abstract] OR "Non Insulin Dependent Diabetes Mellitus"[Title/Abstract] OR "NIDDM"[Title/Abstract] OR "MODY"[Title/Abstract] OR "maturity onset diabetes*"[Title/Abstract] OR "Ketosis Resistant Diabetes Mellitus"[Title/Abstract] OR "insulin independent diabetes*"[Title/Abstract] OR "dm 2"[Title/Abstract] OR "diabetes type 2"[Title/Abstract] OR "diabetes maturity onset"[Title/Abstract] OR "diabetes adult onset"[Title/Abstract] OR "diabetes type II"[Title/Abstract] OR "diabetes type 2"[Title/Abstract] OR "diabetes mellitus type ii"[Title/Abstract] OR "diabetes mellitus stable"[Title/Abstract] OR "diabetes mellitus slow onset"[Title/Abstract] OR "diabetes mellitus noninsulin dependent"[Title/Abstract] OR "diabetes mellitus non insulin dependent"[Title/Abstract] OR	230925	2025/09/27

	"diabetes mellitus maturity onset"[Title/Abstract] OR "diabetes mellitus adult onset"[Title/Abstract] OR "diabetes mellitus type ii"[Title/Abstract] OR "diabetes mellitus type 2"[Title/Abstract] OR "adult onset diabetes*"[Title/Abstract]		
#3	"hypoxia inducible factor 1, alpha subunit"[MeSH Terms]	18980	2025/09/27
#4	"hypoxia inducible factor 1alpha"[Title/Abstract] OR "hypoxia inducible factor 1 alpha subunit"[Title/Abstract] OR "ARNT interacting protein"[Title/Abstract]	10041	2025/09/27
#5	("diabetes mellitus, type 2"[MeSH Terms] OR ("type II diabetes mellitus"[Title/Abstract] OR "type II diabetes"[Title/Abstract] OR "type 2 diabetes*"[Title/Abstract] OR "TIIDM"[Title/Abstract] OR "T2DM"[Title/Abstract] OR "Stable Diabetes Mellitus"[Title/Abstract] OR "noninsulin dependent diabetes*"[Title/Abstract] OR "non insulin dependent diabetes*"[Title/Abstract] OR "Non Insulin Dependent Diabetes Mellitus"[Title/Abstract] OR "NIDDM"[Title/Abstract] OR "MODY"[Title/Abstract] OR "maturity onset diabetes*"[Title/Abstract] OR "Ketosis Resistant Diabetes Mellitus"[Title/Abstract] OR "insulin independent diabetes*"[Title/Abstract] OR "dm 2"[Title/Abstract] OR "diabetes type 2"[Title/Abstract] OR "diabetes maturity onset"[Title/Abstract] OR	185	2025/09/27

	"diabetes adult onset"[Title/Abstract] OR "diabetes type II"[Title/Abstract] OR "diabetes type 2"[Title/Abstract] OR "diabetes mellitus type ii"[Title/Abstract] OR "diabetes mellitus stable"[Title/Abstract] OR "diabetes mellitus slow onset"[Title/Abstract] OR "diabetes mellitus noninsulin dependent"[Title/Abstract] OR "diabetes mellitus non insulin dependent"[Title/Abstract] OR "diabetes mellitus maturity onset"[Title/Abstract] OR "diabetes mellitus adult onset"[Title/Abstract] OR "diabetes mellitus type ii"[Title/Abstract] OR "diabetes mellitus type 2"[Title/Abstract] OR "adult onset diabetes*"[Title/Abstract])) AND ("hypoxia inducible factor 1, alpha subunit"[MeSH Terms] OR ("hypoxia inducible factor 1alpha"[Title/Abstract] OR "hypoxia inducible factor 1 alpha subunit"[Title/Abstract] OR "ARNT interacting protein"[Title/Abstract]))		

Embase

Search	Query	Results	Date
#1	'non insulin dependent diabetes mellitus'/exp	433,928	2025/09/27
#2	'type ii diabetes mellitus':ab,ti,kw OR 'type ii diabetes':ab,ti,kw OR 'type 2 insulin independent diabetes':ab,ti,kw OR 'type 2 diabetes*':ab,ti,kw OR 'tiidm':ab,ti,kw OR 't2dm':ab,ti,kw OR 'stable diabetes mellitus':ab,ti,kw OR 'slow onset diabetes mellitus':ab,ti,kw OR 'noninsulin dependent type 2 diabetes mellitus':ab,ti,kw OR 'noninsulin dependent diabetes*':ab,ti,kw OR 'non insulin dependent diabetes*':ab,ti,kw OR 'non insulin dependent diabetes mellitus':ab,ti,kw OR 'niddm':ab,ti,kw OR 'mody':ab,ti,kw OR 'maturity onset diabetes*':ab,ti,kw OR 'ketosis resistant diabetes mellitus':ab,ti,kw OR 'insulin independent diabetes*':ab,ti,kw OR 'dm 2':ab,ti,kw OR 'diabetes, type 2':ab,ti,kw OR 'diabetes, maturity onset':ab,ti,kw OR 'diabetes, adult onset':ab,ti,kw OR 'diabetes type ii':ab,ti,kw OR 'diabetes type 2':ab,ti,kw OR 'diabetes mellitus, type ii':ab,ti,kw OR 'diabetes mellitus, stable':ab,ti,kw OR 'diabetes mellitus, slow onset':ab,ti,kw OR 'diabetes mellitus, noninsulin dependent':ab,ti,kw OR 'diabetes mellitus, non insulin dependent':ab,ti,kw OR 'diabetes mellitus, maturity onset':ab,ti,kw OR 'diabetes mellitus, ketosis resistant':ab,ti,kw OR 'diabetes mellitus, adult onset':ab,ti,kw OR	370,286	2025/09/27

	'diabetes mellitus type ii':ab,ti,kw OR 'diabetes mellitus type 2':ab,ti,kw OR 'adult onset diabetes*':ab,ti,kw		
#3	'hypoxia inducible factor 1alpha'/exp	41,267	2025/09/27
#4	'member of pas protein 1':ab,ti,kw OR 'hypoxia inducible factor 1alpha':ab,ti,kw OR 'hypoxia inducible factor 1 alpha subunit':ab,ti,kw OR 'arnt interacting protein':ab,ti,kw	1,646	2025/09/27
#5	#1 OR #2	498,893	2025/09/27
#6	#3 OR #4	41,502	2025/09/27
#7	#5 AND #6	629	2025/09/27

Cochrane library

Search	Query	Results	Date
#1	MeSH descriptor: [Diabetes Mellitus, Type 2] explode all trees	26990	2025/09/27
#2	('type II diabetes mellitus' OR 'type II diabetes' OR 'type 2 insulin independent diabetes' OR 'Type 2 Diabetes*' OR 'TIIDM' OR 'T2DM' OR 'Stable Diabetes Mellitus' OR 'Slow Onset Diabetes Mellitus' OR 'noninsulin dependent type 2 diabetes mellitus' OR 'noninsulin dependent diabetes*' OR 'non insulin dependent diabetes*' OR 'Non Insulin Dependent Diabetes Mellitus' OR 'NIDDM' OR 'MODY' OR 'Maturity Onset Diabetes*' OR 'Ketosis Resistant Diabetes Mellitus' OR 'insulin independent diabetes*' OR 'dm 2' OR 'Diabetes, Type 2' OR 'Diabetes, Maturity Onset' OR 'diabetes, adult onset' OR 'diabetes type II' OR 'diabetes type 2' OR 'Diabetes Mellitus, Type II' OR 'Diabetes Mellitus, Stable' OR 'Diabetes Mellitus, Slow Onset' OR 'Diabetes Mellitus, Noninsulin Dependent' OR 'Diabetes Mellitus, Non Insulin Dependent' OR 'Diabetes Mellitus, Maturity Onset' OR 'Diabetes Mellitus, Ketosis Resistant' OR 'Diabetes Mellitus, Adult Onset' OR 'diabetes mellitus type ii' OR 'diabetes mellitus type 2' OR 'adult onset diabetes*'):ab,ti,kw	76229	2025/09/27
#3	MeSH descriptor: [Hypoxia-Inducible Factor 1, alpha Subunit] explode all trees	101	2025/09/27
#4	('member of PAS protein 1' OR 'hypoxia inducible factor 1alpha' OR 'hypoxia inducible factor 1 alpha subunit' OR 'ARNT interacting protein'):ab,ti,kw	126	2025/09/27
#5	(#1 OR #2) AND (#3 OR #4)	5	2025/09/27

WOS

Search	Query	Results	Date
#1	TS=("type II diabetes mellitus" OR "type II diabetes" OR "type 2 insulin independent diabetes" OR "Type 2 Diabetes*" OR "tiide" OR "T2DM" OR "Stable Diabetes Mellitus" OR "Slow Onset Diabetes Mellitus" OR "noninsulin dependent type 2 diabetes mellitus" OR "noninsulin dependent diabetes*" OR "non insulin dependent diabetes*" OR "Non Insulin Dependent Diabetes Mellitus" OR "NIDDM" OR "MODY" OR "Maturity Onset Diabetes*" OR "Ketosis Resistant Diabetes Mellitus" OR "insulin independent diabetes*" OR "dm 2" OR "Diabetes, Type 2" OR "Diabetes, Maturity Onset" OR "diabetes, adult onset" OR "diabetes type II" OR "diabetes type 2" OR "Diabetes Mellitus, Type II" OR "Diabetes Mellitus, Stable" OR "Diabetes Mellitus, Slow Onset" OR "Diabetes Mellitus, Noninsulin Dependent" OR "Diabetes Mellitus, Non Insulin Dependent" OR "Diabetes Mellitus, Maturity Onset" OR "Diabetes Mellitus, Ketosis Resistant" OR "Diabetes Mellitus, Adult Onset" OR "diabetes mellitus type ii" OR "diabetes mellitus type 2" OR "adult onset diabetes*" OR "non insulin dependent diabetes mellitus" OR "Diabetes Mellitus, Type 2")	468,785	2025/09/27
#2	TS=("member of PAS protein 1" OR "hypoxia inducible factor 1alpha" OR "hypoxia inducible factor 1 alpha subunit" OR "ARNT interacting protein" OR "Hypoxia-Inducible Factor 1, alpha Subunit"	23,544	2025/09/27

	OR "hypoxia inducible factor 1alpha")		
#3	#1 AND #2	205	2025/09/27

维普

Search	Query	Results	Date
#1	题名或关键词=2 型糖尿病+diabetes melitus type 2+noninsulin- dependent diabetes mellitus+type2 diabetes+type 2 diabetes mellitus+type 2 diabetic+type ii diabetes+type ii diabetes mellitus+成人发病型糖尿病+糖尿病 2 型+第二型糖尿病+非胰岛素依赖性糖尿病+非胰岛素依赖型糖尿病+二型糖尿病 AND 题名或关键词=缺氧诱导因子 1,α亚基+HIF-1α AND 期刊范围:北大核心期刊,El 来源期刊,SCIE 期刊,CAS 来源期刊	30	2025/09/27

万方

Search	Query	Results	Date
#1	题名或关键词:(2 型糖尿病 OR 成人发病型糖尿病 OR 糖尿病 2 型 OR 第二型糖尿病 OR 非胰岛素依赖性糖尿病 OR 非胰岛素依赖型糖尿病 OR 二型糖尿病) and 题名或关键词:(缺氧诱导因子 1,α亚基 OR HIF-1α)	44	2025/09/27

知网

Search	Query	Results	Date
#1	TKA = ('2 型糖尿病'+'diabetes melitus type 2'+'noninsulin-dependent diabetes mellitus'+'type2 diabetes'+'type 2 diabetes mellitus'+'type 2 diabetic'+'type ii diabetes'+'type ii diabetes mellitus'+'成人发病型糖尿病'+'糖尿病 2 型'+'第二型糖尿病'+'非胰岛素依赖性糖尿病'+'非胰岛素依赖型糖尿病'+'二型糖尿病') AND TKA = ('缺氧诱导因子 1,α 亚基'+'HIF-1α')	109	2025/09/27

Sinomed

Search	Query	Results	Date
#1	"缺氧诱导因子 1,α 亚基"[全部字段:智能] OR "HIF-1α"[全部字段:智能]	35707	2025/09/27
#2	"2 型糖尿病"[全部字段:智能] OR "成人发病型糖尿病"[全部字段:智能] OR "糖尿病 2 型"[全部字段:智能] OR "第二型糖尿病"[全部字段:智能] OR "非胰岛素依赖性糖尿病"[全部字段:智能] OR "非胰岛素依赖型糖尿病"[全部字段:智能] OR "二型糖尿病"[全部字段:智能]	410481	2025/09/27
#3	(#2) AND (#1)	97	2025/09/27
