# Supplementary material for: Peripheral blood HIF-1α levels in type 2 diabetes mellitus and their association with diabetic complications: a systematic review and meta-analysis
Source: Front Endocrinol (Lausanne). 2026 Jul 29;17:1879557. doi: 10.3389/fendo.2026.1879557 (PMC13461533; doi:10.3389/fendo.2026.1879557)
Supplement: Supplementary file 4 [file SupplementaryFile4.pdf]

Agency for Healthcare Research and Quality

		1	2	3	4	5	6	7	8	9	10	11	12	13	14	15	16	17	18	19	20	21	22	23	24	25	26	29	30	31	32	33
		Shao	Zhou	Shao	Shao	E	Zhang	Sun	Han	Jing	Ha	Sun	Yu	Li	Shan	Li	Shen	Liu	Song	Tian	Xiong	Xu	Zhang	Zhu	Bian	Cuore	Gaonka	Gharib	Hu	Ren	Sayed	Shao
1	Define the source of information (survey, record review)	1	1	1	1	1	1	1	1	1	1	1	1	1	1	1	1	1	1	1	1	1	1	1	1	1	1	1	1	1	1	1
2	List inclusion and exclusion criteria for exposed and unexp	1	1	1	1	1	1	1	1	1	0	1	1	1	1	1	1	1	1	1	1	1	1	1	1	1	1	1	1	1	1	1
3	Indicate time period used for identifying patients	1	1	1	1	1	1	1	1	1	1	1	1	0	1	1	1	1	1	1	1	1	1	1	1	1	1	1	1	0	1	1
4	Indicate whether or not subjects were consecutive if not po	0	0	0	0	0	0	0	0	0	0	0	0	0	0	0	0	0	0	0	0	0	0	0	0	1	0	0	0	0	1	0
5	Indicate if evaluators of subjective components of study were masked to other aspects of the status of the participants	1	1	1	1	1	1	1	1	1	1	0	1	1	1	1	1	1	1	1	1	1	1	1	1	1	1	1	1	1	1	1
6	Describe any assessments undertaken for quality assurance	1	1	1	1	1	1	1	1	1	1	1	1	1	1	1	1	1	1	1	1	1	1	1	1	1	1	1	1	1	1	1
7	Explain any patient exclusions from analysis	1	1	1	1	1	1	1	1	1	1	1	1	1	1	1	1	1	1	1	1	1	1	1	1	1	1	1	1	1	1	1
8	Describe how confounding was assessed and/or controlled	1	1	1	1	1	1	1	1	1	0	1	1	0	1	1	1	1	1	1	1	1	1	1	1	1	1	1	1	1	1	1
9	If applicable, explain how missing data were handled in the	1	1	1	1	1	1	1	1	1	1	1	1	1	1	1	1	1	1	1	1	1	1	1	1	1	1	1	1	1	1	1
10	Summarize patient response rates and completeness of data	1	1	1	1	1	1	1	1	1	1	1	1	1	1	1	1	1	1	1	1	1	1	1	1	1	1	1	1	1	1	1
11	Clarify what follow-up, if any, was expected and the percen	0	0	0	0	0	0	0	0	0	0	0	0	0	0	0	0	0	0	0	0	0	0	0	0	0	0	0	0	0	0	0
Total Score		9	9	9	9	9	9	9	9	9	7	8	9	7	9	9	9	9	9	9	9	9	9	9	9	10	9	9	9	8	10	9
